# Debridement Increases Survival in a Mouse Model of Subcutaneous Anthrax

**DOI:** 10.1371/journal.pone.0030201

**Published:** 2012-02-29

**Authors:** Zachary P. Weiner, Anne E. Boyer, Maribel Gallegos-Candela, Amber N. Cardani, John R. Barr, Ian J. Glomski

**Affiliations:** 1 Department of Microbiology, University of Virginia, Charlottesville, Virginia, United States of America; 2 National Center for Environmental Health, Centers for Disease Control and Prevention, Atlanta, Georgia, United States of America; Loyola University Medical Center, United States of America

## Abstract

Anthrax is caused by infection with *Bacillus anthracis*, a spore-forming Gram-positive bacterium. A major virulence factor for *B. anthracis* is an immunomodulatory tripartite exotoxin that has been reported to alter immune cell chemotaxis and activation. It has been proposed that *B. anthracis* infections initiate through entry of spores into the regional draining lymph nodes where they germinate, grow, and disseminate systemically via the efferent lymphatics. If this model holds true, it would be predicted that surgical removal of infected tissues, debridement, would have little effect on the systemic dissemination of bacteria. This model was tested through the development of a mouse debridement model. It was found that removal of the site of subcutaneous infection in the ear increased the likelihood of survival and reduced the quantity of spores in the draining cervical lymph nodes (cLN). At the time of debridement 12 hours post-injection measurable levels of exotoxins were present in the ear, cLN, and serum, yet leukocytes within the cLN were activated; countering the concept that exotoxins inhibit the early inflammatory response to promote bacterial growth. We conclude that the initial entry of spores into the draining lymph node of cutaneous infections alone is not sufficient to cause systemic disease and that debridement should be considered as an adjunct to antibiotic therapy.

## Introduction

The disease anthrax is caused by infection with *Bacillus anthracis*, a Gram-positive bacterium whose dormant spores can infect most warm-blooded hosts [1], [2]. There are three different forms of anthrax that are defined by the route by which spores are introduced into the host: by inhalation, gastrointestinaly, and cutaneously. Cutaneous infections account for ∼95% of all human cases and carry the lowest morbidity and mortality for anthrax infections, resulting in 1% mortality upon treatment with antibiotics and up to 20% mortality when untreated [3]. Eleven of the twenty-two cases of anthrax associated with the intentional release *B. anthracis* spores through the U.S. mail system in 2001 were cutaneous infections, demonstrating the propensity of this bacterium to cause soft tissue infections [4].

Soft tissue debridement consists of surgically removing the infected and/or necrotic tissue to promote healing and physically eliminate the organisms infecting the tissue [5]. Debridement is typically used, along with appropriate antibiotics, in the management of necrotizing fasciitis, gangrene, and soft-tissue infections caused by a wide range of organisms including *Staphylococcus aureus*, *Clostridium perfringens*, and other pathogens that are refractory to antibiotic treatment [6]. In these situations the infected tissues are sacrificed to prevent the rest of the body from becoming overwhelmed by the infection. Soft tissue infections by *B. anthracis* demonstrate similarities to these infections, including extensive tissue damage, necrotizing fasciitis, and having the potential to cause lethal systemic infections [7]; thus debridement has been examined as a means to reduce overall mortality [8], [9], [10]. The two dominant views regarding its use for anthrax polarize around whether surgery increases the risk of releasing bacteria into the circulatory system versus removing the foci of infection to prevent future spread of bacteria. Nonetheless, debridement was used as a treatment for patients that injected *B. anthracis*-contaminated heroin subcutaneously (“popping”) or intramuscularly [7], [11]. We propose that the debate about when and if to apply debridement to anthrax persists because the question has never been addressed with a controlled experimental model in which one experimental variable can be altered to assess cause and effect. The goal of this study was to develop and substantiate an animal model of debridement for anthrax to provide a broadly accessible tool for the production of experimental evidence to guide the use of debridement in the treatment of *B. anthracis* infections.

*B. anthracis* virulence is increased through the secretion of a tripartite exotoxin and encapsulation with a poly-D-glutamic acid capsule [12]. The tripartite exotoxin consists of protective antigen (PA), lethal factor (LF), and edema factor (EF) [13]. PA in combination with LF is referred to at lethal toxin (LT), and PA combined with EF is referred to edema toxin (ET). When considering the clinical implications of *B. anthracis* exotoxins, of particular importance is the observation that, even with effective antibiotic treatment that eliminates detectible live bacteria, individuals demonstrating systemic signs of anthrax have a poor prognosis [14]. This observation is often attributed to the lingering effects of these exotoxins and must be considered when assessing the timing and efficacy of debridement [2].

The most widely recognized model for *B. anthracis* dissemination through the host is referred to as the Trojan horse model [15], [16]. In this model, dormant spores are phagocytosed by resident phagocytes and then transported to the draining lymph node where they germinate and grow. Once the lymph node is overwhelmed by bacterial growth, the bacteria then gain access to the circulatory system via the efferent lymphatics. Most of the data that support this model were produced with the aim of deciphering the dynamics of inhalation anthrax. It is unclear how much of this model applies to other versions of anthrax. Multiple studies indicate that dissemination unfolds similarly after the draining lymph nodes have been infected irrespective of the initial site of infection [17], [18]; implying that differences in the lethality observed between cutaneous, gastrointestinal, or inhalation anthrax are manifest upstream of the lymph nodes.

In the course of developing the debridement model in mice it became apparent that the accumulating data had direct implications on the application of the Trojan horse model to subcutaneous infections. We hypothesized, based on the inhalation Trojan horse model, that *B. anthracis* spores injected subcutaneously will quickly enter the draining lymph node and that the spores that enter the lymph node early after inoculation are the founding population of bacteria of the disseminated infection. This hypothesis was tested by utilizing a luminescence-based mouse ear model of subcutaneous anthrax coupled with debridement to achieve delivery of a pulse of spores to the cervical lymph nodes (cLN) draining the injection site. By removing the initial site of inoculation the resultant infection is limited to those bacteria that have entered the cLN during the time interval from injection to excision of the injection site.

In this study we report that debridement of the site of subcutaneous inoculation of *B. anthracis* spores in the mouse ear up to 48 hours post-infection significantly increased the likelihood of host survival. This observation questions the universality of the Trojan horse dissemination model, which implies that the early transit of *B. anthracis* into the lymph node is an essential step towards systemic infection. In this subcutaneous model, the early delivery of spores to the cLN was not sufficient to establish disseminated infection. Instead bacterial growth at the initial site of subcutaneous infection was necessary; an observation that does not support the Trojan horse model in subcutaneous anthrax. We propose that the initial site of cutaneous infection supplies bacteria and exotoxins to the lymph node at a later time to cause the disseminated infection. This mouse model of subcutaneous infection experimentally demonstrated that debridement improves the outcome of subcutaneous *B. anthracis* infections by limiting the influence of bacteria within the initial site of infection and thus should be further explored experimentally as an adjunct to antibiotic therapy.

## Results

**Figure 1:**
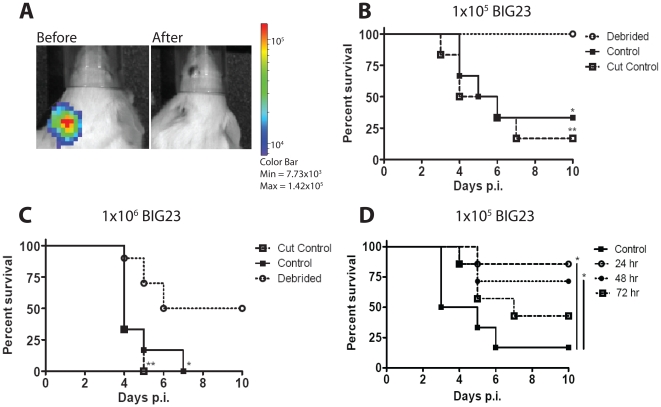
Debridement significantly increases mouse survival from subcutaneous *B. anthracis* infection. (A) Black and white photos of a single A/J mouse infected with 1×10^5^ BIG23 spores in the left ear overlaid with false-color representation of photon emission intensity as indicated by the scale on the right in p/s/cm-2/sr. At 12 hours post injection luminescent infected tissue (left photo- before) was removed (debrided) to eliminate all bacterial luminescence (right photo- after). (B to D) BIG23 spores were injected subcutaneously into the left ear of 4–12 week old A/J mice. Control mice either had their left ear unperturbed (control) or non infected tissue on the same ear was removed (cut control). Mice were then monitored for infection and dissemination using in vivo bioluminescent imaging. Mice were monitored for a total of ten days post-infection (p.i.), until luminescence was detected in a major organ, or mouse appeared moribund (indicating imminent death). (B) Mice were inoculated with 1×10^5^ spores of BIG23. Luminescent tissue was debrided at 12 hours p.i. Survival data from a total of seven mice from three independent experiments were analyzed with the log rank test to determine significant differences in survival between debrided and control mice (*, p = 0.0108), and debrided and cut control mice (**, p = 0.0021). There was no statistical difference between control and cut control mice. (C) Mice were inoculated with 1×10^6^ spores of BIG23. Luminescent tissue was debrided at 12 hours p.i. Survival data was analyzed with the log rank test on a total of ten debrided mice and seven mice each for the control groups from three independent experiments to determine significant differences in survival between debrided and control mice (*, p = 0.0109) and debrided and cut control mice (**, p = 0.0026). There was no statistical difference between control and cut control mice. (D) Mice were infected with BIG23 as described above, but debridement of luminescent tissue was performed at 24, 48, or 78 hour p.i. Survival data was analyzed with the log rank test on a total of seven mice from each experimental group, with the exception of the control mice that totaled six, from three independent experiments to determine significant differences from control mice. The survival of mice debrided at 24 hours (*, p = 0.0110) and 48 hours (*, p = 0.0477) p.i. was significantly increased. The survival of mice debrided at 72 hours p.i. was not significantly different than control mice (p = 0.1734).

Debate about the appropriate application of debridement to soft tissue infections by *B. anthracis* has been hampered by lack of experimental evidence to indicate or contraindicate its use. We thus sought to develop a mouse model for debridement. To achieve this goal spores from a *B. anthracis* Sterne strain that produces light during vegetative growth within a host (BIG23) were injected subcutaneously in the ear of C5-deficient A/J mice. Infection of A/J mice with non-capsulated toxigenic *B. anthracis* Sterne strain is a widely used animal model for anthrax that recapitulates many characteristics of infections caused by encapsulated toxigenic *B. anthracis* strains such as the Ames strain [19], [20], yet poses less risk to researchers, and thus makes the model described here broadly accessible to researchers without BSL3 facilities. The ear was chosen as the anatomic site of infection for our studies because the ear architecture allows efficient debridement, its translucence permits greater sensitivity when detecting bacterial growth by light emission, and has been frequently used for modeling infectious diseases [18], [21], [22], [23], [24]. Bioluminescent *B. anthracis* strains were applied in order to qualitatively identify the initial site of infection, determine whether all infected tissues were removed during the debridement procedure, and allow the assessment of the dissemination of bacteria over time. Initial sites of infection were identified by bacterial luminescence at 12, 24, 48, and 72 hours and debrided by notching the ear to efficiently remove the initial site of infection (Figure 1A); similar to general mouse husbandry ear tagging procedures. Control infected mice had a section of ear removed that did not contain the infection, or as a second control, no portion of the ear was removed. Debridement at 12 hours significantly protected mice from subcutaneous infection with two different doses of spores (Figure 1B and 1C). All mice that were inoculated with 1×10^5^ spores and debrided survived, whereas 67% of the control groups succumbed to systemic infections (Figure 1B). Similarly, in mice inoculated with 1×10^6^ spores (approximately 100 LD_50_s), 50% of the debrided mice survived, while all mice from control groups succumbed to systemic infections (Figure 1C). Debridement of the ear significantly decreased mortality when performed at 12, 24, and 48 hours post-infection, but lost significance after 72 hours post-infection (Figure 1D). We conclude that debridement significantly protected mice from subcutaneous infection if performed within 48 hours of subcutaneous inoculation.

**Figure 2:**
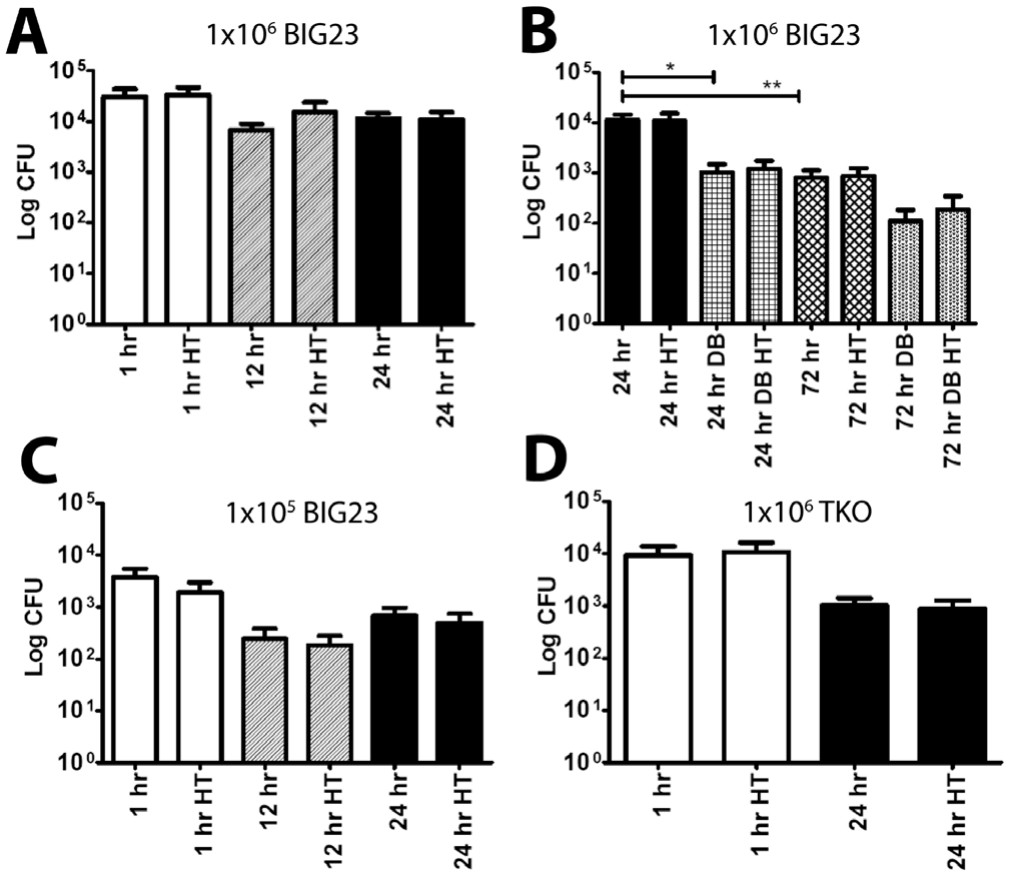
*B. anthracis* spores are found in the draining cervical lymph node early in infection. (A–C) BIG23 spores were inoculated subcutaneously into the left ear of 4–12 week old A/J mice. At the indicated times cervical draining lymph nodes were removed and CFU derived from vegetative cells verses spores were differentiated by quantifying samples with and without heat treatment (HT). Bars denote the mean and error bars represent the standard error of the mean. Significance was determined using Student's T-est. (A) Quantification of bacteria within the draining lymph node at 1, 12, and 24 hours post-infection (p.i.) from mice inoculated with 1×10^6^ spores. There were no significant differences with or without heat treatment or between different time points using a total of six mice in three independent experiments. (B) Quantification of bacteria within the draining lymph node at 24 and 72 hours p.i. with and without debridement (DB) performed at 12 hours after inoculation with 1×10^6^ spores. Significant differences were observed in a total of six mice in three independent experiments between 24 hr and 24 hr DB (*, p = 0.0144) and 24 hr and 72 hr (**, p = 0.0066). There were no significant differences between heated and unheated samples at either time with or without debridement. (C) Bacteria within the lymph node were quantified similarly to A at 1, 12, and 24 hours p.i., but with an inoculation of 1×10^5^ spores in a total of six mice in three independent experiments. There were no significant differences with or without heat treatment. (D) Bacteria within the lymph nodes were quantified similarly to A at 1 and 24 hours p.i., but instead, 1×10^6^ spores of the mutant strain that produced no exotoxins (TKO) were inoculated in a total of six mice in three independent experiments. There were no significant differences with or without heat treatment.

There are multiple mechanisms by which debridement could promote survival, one of which is the prevention of bacterial dissemination from the site of inoculation to the draining lymph node. We thus questioned the timing of entry of *B. anthracis* into the draining lymph node post-inoculation and if bacteria had indeed entered the draining lymph nodes during the interval between inoculation and debridement as is predicted by the Trojan horse model of infection. Accordingly, at 1, 12 and 24 hours post-infection the cervical draining lymph nodes were removed and bacterial colony-forming units (CFU) were measured for both the total number of bacteria (spores+vegetative cells) by plating without heat treatment or for spores-only by heating the samples to 65°C for 20 minutes to kill all germinating spores and vegetative bacteria, and then plating for CFU [25]. Independent of the initial dose, approximately 3–4% of injected spores arrived in the draining lymph nodes as early as one hour after injection (Figure 2). The number of spores in the lymph node remained constant, and no outgrowth occurred as late as 24 hours after infection (Figure 2A). To test the effect of debridement on subsequent bacterial loads, lymph nodes were removed at 24 and 72 hours from mice that underwent debridement at 12 hours or non-debrided control mice. At 72 hours post-injection the CFU had dropped significantly by over a log and as at earlier time points, all CFU were comprised of spores (Figure 2B). With debridement there were significantly fewer spores in the cLN at 24 hours post-infection (p = 0.0144), and insignificantly fewer spores at 72 hours post-infection (p = 0.0066) (Figure 2B). These findings were unaffected by the dose of spores administered, however the magnitude of measured CFU was proportionally lower with the lower inoculum dose (Figure 2A and 2C). At no time were CFU isolated from the contralateral lymph nodes draining the uninoculated right ear (data not shown). Similar cLN CFU were observed in infections using spores from a *B. anthracis* strain that had PA, LF, and EF production eliminated (TKO) (Figure 2D), suggesting that the above findings are spore mediated and not LT or ET dependent. In total these data suggest that at the time of debridement at 12 hours post-inoculation spores can be found in the draining lymph nodes and thus these spores are typically not sufficient to cause systemic infections since debridement effectively increased the likelihood of mouse survival.

The clinical difficulty of treating anthrax, even after the bacteria have been effectively controlled with antibiotics, has been attributed to residual exotoxin activity within the host. Likewise, *B. anthracis* exotoxins have been reported to modulate chemotaxis and activation of host innate immune cells [26], [27], [28], [29]. We thus questioned whether exotoxins were present at the time of debridement, since they could be locally influencing the chemotaxis of potential Trojan horse cells, altering the activation state of innate immune defenses, or contributing to overall mortality even after the bacteria were surgically removed. At 12 hours, the earliest time of debridement, 458±216 pg LF was isolated from the ear, 28±10 pg in the draining lymph node, and 476±82 pg/ml in the serum of mice that received 1×10^6^ BIG23 spores subcutaneously (Table 1). When TKO spores were co-inoculated with purified LT (100 ng LF+650 ng PA), the LF distributed into the ear, lymph nodes, and serum similarly to the LF actively produced by the exotoxin-producing BIG23; with relatively equal proportions of the total LF distributed to the ear and serum and approximately one-twentieth of the quantity found in the cLN as in the ear and serum (Table 1). These LF concentrations were assessed using a highly sensitive mass spectrometry-based assay for LF enzymatic activity that was originally designed to assess LF concentrations in serum [30], but was adapted here to study solid tissues. To date this is the most sensitive assay for detecting any of the components of the *B. anthracis* exotoxins and may reflect total exotoxin production since the individual exotoxin components are coordinately regulated [31]. Notably, the majority of debrided mice did not succumb to infection, even with measurable circulating exotoxins, implying that these concentrations of LF alone are not sufficient to mediate death. Likewise when TKO spores were used to initiate infection, a similar number of spores were found in the cLN as wild-type Sterne strain bacteria during the same time period (Figure 2D). This suggests that spore entry into the cLN is not affected by LT or ET, counter to what may have been predicted based on published cell culture analyses that demonstrated exotoxin-based alterations in phagocyte activation and chemotaxis towards lymph nodes [27], [32], [33], [34], [35].

**Table 1:** Lethal Factor is measurable in the ear, draining cervical lymph nodes and serum at the time of debridement 12 hours after spore inoculation.

Strain	Ear^C^	cLN^C^ ^D^	Serum/ml^C^
BIG23^A^	458±216	28±10	476±82
TKO^B^	1592±681	49±13	2354±223

A Injection of 1×10^6^ luminescent 7702 (BIG23) spores subcutaneously into left ear of 3 A/J mice.

B Injection of 1×10^6^ triple exotoxin knockout (TKO) spores with 100 ng LF plus 650 ng PA into left ear of 3 A/J mice.

C LF detected by anatomic locations (pg/tissue ± SE) 12 hours post inoculation. Average of three separate experiments with three mice.

D Cervical lymph node.

**Figure 3:**
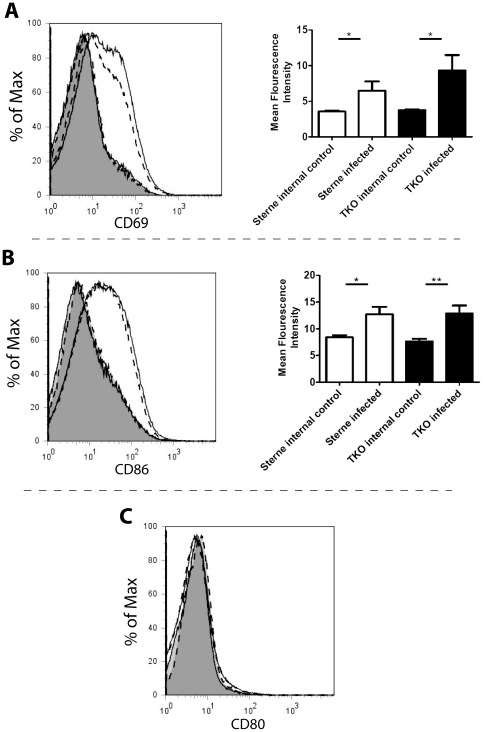
Presence of lethal factor in cervical draining lymph node does not alter cellular activation. Mice were infected with 1×10^6^ Sterne strain or TKO spores subcutaneously in the left ear. At 24 hours cLN were taken from mice including: Sterne infected mice (solid line non-shaded) along with control non-infected contralateral lymph nodes from Sterne infected mice (solid line shaded), and TKO infected (dashed line non-shaded) and control non-infected contralateral lymph nodes from TKO infected mice (dashed line shaded). cLN were homogenized and cells were stained for flow cytometry. Flow plots were gated for single event live CD45 positive cells. (A) Left- a representative histogram comparing expression of CD69 in draining cLN. Right- The mean fluorescence intensity of lymph nodes draining either Sterne (*, p = 0.0464) or TKO (*, p = 0.0256) infections from seven mice was significantly higher than contralateral control lymph nodes as determined using Student's T test. (B) Left- a representative histogram comparing expression of CD86 in draining cLN. Right- The mean fluorescence intensity of lymph nodes draining either Sterne (*, p = 0.0119) or TKO (**, p = 0.0063) infections from seven mice was significantly higher than contralateral control lymph nodes as determined using Student's T test. (C) A representative comparison of CD80 expression in draining cLN. The mean fluorescence intensity of lymph nodes draining either Sterne or TKO infections from seven mice was not significantly higher than lymph nodes that were not.

Since exotoxins were present at the earliest time of debridement, we next asked whether they had any measurable effect on innate immune cell activation within the draining lymph nodes. Accordingly, spores from *B. anthracis* Sterne or the TKO mutant were injected subcutaneously into the left ear of mice at a concentration of 1×10^6^ spores and at 24 hours cLN were removed, homogenized, and stained for flow cytometric analysis. Expression of the activation markers CD69, CD80 and CD86 were chosen as measures of activation since previous publications have demonstrated that these cell markers are down regulated by *B. anthracis* exotoxins [36], [37], [38]. Specifically, CD80 and CD86 are down regulated in dendritic cells [37], [39], and CD69 is down regulated in response to LT in T cells at 24 hours [36]. Flow cytometry revealed that expression of CD69 and CD86, but not CD80, were significantly up-regulated in mice that had been infected with Sterne or TKO spores as compared to internal control lymph nodes (Figure 3A to C). The degree of up-regulation was the same regardless of the ability of bacteria to produce exotoxin. cLN analyzed at 12 hours post-infection demonstrated no increase in CD69, CD80, or CD86 under any conditions (data not shown). These findings suggest that exotoxins are not present in the lymph node in sufficient quantities to alter cellular response to inflammatory stimuli in the first 24 hours of *B. anthracis* infection.

## Discussion

Early events in *B. anthracis* infections are crucial in determining the ultimate outcome of infection. Antibiotic treatment can eliminate most vegetative bacteria growing in a host, yet frequently the sequelae of infection exacerbated by lingering exotoxins still lead to death [2]. It is therefore vital that the early events in *B. anthracis* infections are better understood such that properly conceived therapeutic interventions can be administered to promote survival. The studies described above explored early events in subcutaneous *B. anthracis* infections and support the following major conclusions: 1) debridement can effectively reduce the lethality of subcutaneous infections in a mouse model of infection, and 2) early delivery of spores into the draining lymph nodes during subcutaneous infections is not sufficient to cause disseminated disease.

In addition to appropriate antimicrobial therapy, debridement has been an effective method in the management of certain soft tissue infections for over a century [5]. The data derived from the mouse model of subcutaneous infection support the consideration of debridement as therapy for some cases of subcutaneous anthrax, since survival was significantly improved in mice debrided within 48 hours of infection; though it should be noted that debridement at 72 hours post-infection trended towards greater protection as well. These findings may have particular relevance to infections initiated by subcutaneous introduction of illicit drugs, such as those documented in Scotland in 2010 and Norway in 2000 [7], [40]. To our knowledge this is the first controlled experimental evidence to support debridement in anthrax associated with soft tissues. Although there are important caveats to using a mouse model of infection, advantages offered by murine models of infection include the availability of genetically manipulated mice, an extensive array of immunological reagents, and the potential for producing data of greater statistical strength granted by their high reproduction rate and small body size. Subsequent corroboration of that which was learned in mice to studies in non-human primates or retrospective studies where debridement was applied to human anthrax would ultimately be necessary to demonstrate practical utility in human anthrax. Likewise, it should not be overlooked that the model system applied in these studies utilizes a BSL2 *B. anthracis* Sterne strain that does not produce capsule. The lack of capsule production has been demonstrated to alter aspects of pathogenesis in mouse models of infection [18], [41]. Some strains of capsulated exotoxin-producing *B. anthracis* do not require exotoxins to maintain full virulence in subcutaneous infection, such as the Ames strain [42], yet others such as UT500 do require exotoxin production for full virulence [43]. Thus, it is unknown how bacterial capsule production may affect the observations reported here, but should be addressed by future research.

As proposed by the Trojan horse model of infection, spores that enter the lymph nodes at the earliest stages of infection eventually cause the disseminated infection [16]. This model would predict that removal of the site of infection by debridement after spores had entered the draining lymph nodes should have minimal effect on the outcome of infection, since it is these spores that are most important to establishing a lethal infection. This, however, was not observed; instead, mice were more likely to survive with debridement. A similar sequence of events would then unfold in the lymph node, such that when its resistance thresholds are overcome by bacteria and/or exotoxins, the bacteria and exotoxins then spill into the lymphatics and blood to cause bacteremia and/or toxemia leading to eventual death. Indeed, *B. anthracis* exotoxins were detected at the site of infection, in the circulatory system and in the draining lymph nodes at 12 hours post-infection as described above. Thus debridement may improve outcomes by simultaneously removing bacteria and eliminating the source of exotoxins that disperse to tissues not containing bacteria. That which defines the thresholds controlling the infection locally has not been clearly defined, but likely includes effector cells of the innate immune response; implying that the immune status of the host is vital in defining the final outcome of infection.

Considering the Trojan horse model, the improved survival with debridement was unexpected, especially considering that the primary inoculation dose would have been lethal otherwise. However, the totality of results presented above for the cLN provide evidence that supports a different mechanism of dissemination in subcutaneous infections. Approximately 4% of the spores that were inoculated subcutaneously could be isolated from the draining cervical lymph node within 1 hour of infection. The number of spores in the lymph node remained unchanged for the first 24 hours and then decreased by more than a log by 72 hours. At no time were vegetative heat-sensitive bacteria detected in the cLN during this time course. Spores are generally considered resistant to immune cell killing until they germinate [44], thus the constant number of spores detected over the first 24 hours in undebrided mice may represent either a static population of spores or a dynamic balance achieved by the influx of new spores countered by germination and rapid killing of nascent vegetative bacteria. A significant decrease in spores was detected in the cLN at 24 hours after debridement at 12 hours, which supports the second scenario, where total spore counts represent a balance between influx and destruction.

Another unexpected finding was that early exotoxin production had no effect on the ability of spores to enter or persist in the lymph nodes or activate immune cells. The Sterne strain and the TKO mutant showed no significant differences in bacterial CFU in the lymph nodes at any time point or in their activation of the cells within the lymph nodes despite evidence for production of exotoxins in the tissues by the Sterne strain at equivalent times post-infection. Of particular relevance is that ET has been proposed to promote the chemotaxis of phagocytes towards lymph nodes [35], [39], [45] and that LT has been extensively characterized for its ability to modulate the immune system [27], [32], [33]. For these reasons, we predicted that a greater number of spores from an exotoxin-producing strain would be found in the lymph nodes and that there would be a less robust immune response characterized by the expression of fewer activation markers by cells within the lymph nodes. In the context of a subcutaneous infection, our data do not support these roles for the exotoxins, especially since measurable exotoxin was present at the time of debridement at 12 hours post-infection. The cause of this discrepancy is unclear but may reflect the fact that the quantities and timing of the addition of exotoxins in previously published systems may not accurately represent those during an active infection *in vivo*. Indeed, the concentrations of LF detected in the model described above are orders of magnitude lower than those measured in the serum of Rhesus macaques two to four days post-inhalation of spores [30]. The serum of these monkeys contained LF in the hundreds of nanogram per milliliter range, which is closer to the concentration of LF typically used in cell culture assays assessing the affects of LT on cellular functions. It thus may follow that the reported effects of *B. anthracis* exotoxins on cells of the immune system better represent phenomena in later stages of infection rather than the early stages of infection that were explored in this study. In total these data suggest that during the first day post-inoculation the host response in the lymph nodes, 1) is not affected by the presence or absence of the anthrax exotoxins as indicated by the measures described above, and 2) is effective at eliminating *B. anthracis*.

The observation that the lymph nodes are the site of considerable host defense is not surprising in view of the fact that they consist of a diverse collection of immune effector cells. It does however provide some doubt that sufficient quantities of spores are carried by Trojan horse phagocytes to the lymph node at the early stages of infection to ultimately overwhelm the lymph node defenses. All indications from this study are that the lymph node is an effective bulwark against systemic infection unless the initial site of infection continues to feed bacteria and/or spores and exotoxins into the lymph node. Thus the early movement of spores into the lymph nodes in subcutaneous infection, either by phagocyte transport or as extracellular bacteria, is unlikely to have a significant effect on the ultimate outcome of the infection.

In summary, this study describes the first animal model system to experimentally assess debridement as a method of treating subcutaneous anthrax. In addition, the debridement model was readily applied to exploring basic aspects of subcutaneous *B. anthracis* infection. In particular it was effective at assessing the most common model explaining early stages of *B. anthracis* infection, whereby spores are seeded into the draining lymph node where they grow and eventually cause disseminated disease. The data acquired from this analysis suggest that spores initially entering the draining lymph nodes have less of an influence on the ultimate disseminated disease than those spores that continue to reside and grow at the initial site of inoculation.

## Materials and Methods

### Ethics Statement

All mouse husbandry and manipulation were performed following protocols approved by the University of Virginia Animal Care and Use Committee (protocol #3671) conforming to AAALAC International accreditation guidelines. When at all possible we have strived to replace the use of animals in our studies with *in vitro* or non-invasive assays, reduce the number of animals utilized, and refine our use of animals to minimize their suffering and maximize the data extracted from each experiment.

### Bacterial Strains and growth conditions

*B. anthracis* Sterne strain 7702 was obtained from BEI Resources (Manassas, VA). The *B. anthracis* Sterne 7702 strain with deletion of the *pagA*, *lef*, and *cya* genes (referred to in the text as the triple exotoxin knockout or TKO) was graciously provided by Dr. Scott Stibitz from the Center for Biologics Evaluation and Research, Food and Drug Administration [46]. BIG23 was constructed by integrating plasmid pIG6-19, a kind gift from Michele Mock at the Pasteur Institute, into *B. anthracis* strain 7702 using previously described conjugative methods [18]. The resultant BIG23 expressed the *luxABCDE* genes from *Photorhabdus luminescens* under the control of the protective antigen promoter, therefore the vegetative cells of this strain as luminescent when growing with the host. Spores of each *B. anthracis* strains were generated on NBY 5 µg/ml erythromycin agar plates with subsequent purification on an Omnipaque (GE Healthcare, Inc., NJ) gradient as previously published [18].

### Mouse infection

4–12 week old female A/J mice were obtained from Jackson labs, or bred in specific pathogen-free conditions within vivaria at UVA. Mice were anesthetized with 3% isofluorane (Piramal Healthcare, Andhra Pradesh, India) mixed with oxygen using an Isotec 5 vaporizer (Absolute Anesthesia, Piney River, VA). Purified *B. anthracis* spores at a dose of 1×10^6^ or 1×10^5^ spores in 10 µl PBS (Invitrogen, Carlsbad, CA) were injected subcutaneously in the left ear using a 0.5 cc insulin syringe as previously published [18].

**Lymph node isolation:** At designated time points infected mice were euthanized and lymph nodes were surgically removed and placed in sterile PBS on ice. The left cervical lymph node from the infected side of the mouse was harvested as the experimental sample and the right lymph node was harvested as an internal control. The location of the lymph node draining the subcutaneous compartment of the left ear was established by injecting India ink in the same manner as the spore inoculum and then identifying which lymph node turned black [47]. Only one cervical lymph node turned black and subsequent analysis found CFU solely within this lymph node. No other cervical lymph nodes turned black or contained CFU at early stages of infection, suggesting that only the lymph node that directly drains the subcutaneous compartment was infected by *B. anthracis* and not other lymph nodes or surrounding tissues.

### Tissue CFU determination

Lymph nodes were isolated as previously described, immediately stored on ice, and dissociated by grinding between two glass slides. Cells were lysed in distilled water for 15 minutes on ice. Serial dilutions were performed in 1× sterile PBS on ice. Samples were plated onto BHI agar plates, and incubated at 37°C overnight. Determination of dormant spores verses vegetative bacilli was established by placing samples in a 65°C water bath for twenty minutes to kill vegetative bacteria but leave dormant spores unaffected.

### Flow cytometry staining

Single cell suspensions of individual lymph nodes were resuspended in 100 µl flow cytometry staining buffer (1×PBS 3% FBS 0.05% Sodium Azide). Fc receptor-blocking antibody (CD16/32, eBioscience San Diego, CA) was added and incubated on ice for thirty minutes. Samples were centrifuged and resuspended in 100 µl flow staining buffer. Antibodies against specific cell surface markers were added, incubated for one hour on ice, and then washed. Samples were then fixed overnight with 4% paraformaldehyde at 4°C. Antibodies used were: anti-Ly6C FITC (RB6-8C5, BD Biosciences), anti-CD86 PE (PO3.1, eBioscience San Diego, CA), anti-CD11b APC (M1/70, eBioscience), anti-MHC II PacBlue (M5/114.15.2, eBiosciences), anti-CD80 PE Cy5 (16-10A1, eBioscience), anti-CD69 PE Cy7 (H1.2F3, eBioscience), and anti-CD45 Alexa780 (30-F11, eBioscience). Live/Dead staining was performed with fixable Aqua Dead Cell Stain kit (Invitrogen).

### Infection monitoring and luminescent imaging

In order to monitor the progression of infections using luminescent BIG23, mice were anesthetized using 3% isoflourane mixed with oxygen from the XGI-8 gas anesthesia system supplied with a Xenogen IVIS Spectrum. Images were acquired as previously reported and analyzed using Living Image software (version 2.50.1, Xenogen) [18]. Once any detectible luminescent signal was detected in a vital organ, or a mouse appeared moribund, mice were euthanized per animal use protocol and scored as dead in the survival assays.

### Measurement of Lethal Factor concentration in tissue samples

Mice were infected with 1×10^6^ spores of BIG23 spores or TKO spores with 100 ng LF (*B. anthracis* recombinant exotoxin, BEI Resources)+650 ng PA (*B. anthracis* recombinant exotoxin, BEI resources) subcutaneously in the left ear. At 12 hours ears, draining cervical lymph nodes, and blood were removed from infected mice and samples were placed on ice. Then 100 µl of cell lysis cocktail consisting of 0.2% Triton X-100 (Promega), 0.56 mg/ml Pefablock (AppliChem. Darmstadt, Germany), 3.125 mg/ml 6-aminohexanoic acid (Spectrum chemical. Brunswick, NJ), 0.3125 mg/ml Antipain (VWR. West Chester, PA), and 22 µg/ml of E64-D (Cayman Chemical. Ann Arbor, MI) in PBS were added to samples. Samples were then homogenized using kontes pellet pestles (Fisher Scientific. Waltham, MA). Samples then sat on ice for 1 hour. Samples were then spun down and supernatants were removed and stored at −80 C. Blood samples were allowed to clot for 20 minutes then spun down to collect serum, which was then stored at −80°C. Quantification of LF was performed using matrix assisted laser desorption/ionization (MALDI) time of flight (TOF) mass spectrometry (MS) to detect peptide cleavage products generated by LF as previously described [30].

### Statistical Analysis

All statistical analysis and was performed using GraphPad Prism software (version 5, GraphPad Software, San Diego, CA) with log rank test for survival studies, and unpaired t-test for differential CFU in draining lymph node.
